# Memory and menopause: an unsolved puzzle

**DOI:** 10.18632/aging.101596

**Published:** 2018-10-15

**Authors:** Alesia V. Prakapenka, Heather A. Bimonte-Nelson

**Affiliations:** 1 Department of Psychology, Arizona State University, Tempe, AZ 85287, USA; 2 Arizona Alzheimer’s Consortium, Phoenix, AZ 85014, USA; 3 Barrow Brain Tumor Research Center, Barrow Neurological Institute, Phoenix, AZ 85013, USA

**Keywords:** menopause, hormone, estrogen, progesterone, memory, cognition, aging

Menopause is a natural part of life that impacts every woman as aging ensues. Clinically, menopause in women is defined by a halt in menses for at least one year, as ovarian function and ovulation are attenuated [1]. The road to menopause can be transitional and natural, or surgical if a woman undergoes surgical removal of parts of her reproductive system (e.g., the ovaries). In both transitional and surgical menopause, there is ultimately a marked decrease in ovarian-derived circulating hormone levels. These steroid hormones, including estrogens and the natural progestogen, progesterone, not only maintain and support reproductive function, but also play key roles in numerous other bodily functions and systems, including brain-mediated functions such as cognition [2]. Indeed, these two classes of steroid hormones are present, and can even be synthesized, in the brain, as are respective receptors [3]. With menopause, a woman can experience a variety of symptoms that impact quality of life. Exogenously administered hormone therapy (HT) can be clinically used to prevent many undesirable menopause symptoms (e.g., vasomotor and sleep disturbances, vaginal and vulvar atrophy, osteoporosis). Hormone therapy is not currently approved or recommended for aiding cognitive symptoms associated with menopause, as clinical data available to date do not definitively support HT benefits on cognition or dementia outcomes [4]. Of note, amid the collection of imperfectly-fitting puzzle pieces that represent the uncertainty regarding whether HT yields positive cognitive effects, there have been exciting and illuminating scientific discoveries that have guided our direction as a field, allowing sections of the completed puzzle picture to emerge. If we think across bodily systems in an interdisciplinary fashion, if we think creatively, and if basic scientists and physicians work together, our drive forward will continue with an escalating momentum, the puzzle will yield a more complete picture, and marked enrichments in the standard of care for women on the path to menopause, in menopause, and in post-menopause will ensue.

Studies over the last several decades indicate that estrogens and progestogens can impact learning and memory and related brain substrates, although as a field we are still left with missing puzzle pieces of information regarding the optimal hormone milieu parameters that yield consistent and predictable beneficial impacts on brain and cognitive health. Part of the difficulty in predicting the role of HT in brain health during aging is that by the time decisions need to be made about menopause trajectory and HT, each woman has been living a life with a rich and complex history which has undoubtedly included a particular hormone exposure timeline abound with endogenous and exogenous hormone exposures. We know a lot about the effects of some hormone exposures, but not enough yet. We know that particular hormone exposures and profiles can pivot the impact of HT from potentially positive to neutral, or to negative. But which specific hormones, administered in which way, during which time frames, mapped onto which hormone background and history? Our job as scientists is to discover which historical and current lifetime events and exposures are critical enough to pivot the efficacy of HT, and how certain events or exposures act independently as well as interactively to yield particular outcomes – which events are important enough to earn a piece in the puzzle so that the complete picture of menopause and HT can be optimized and realized?

While it is critically important to systematically evaluate in women putative events that may be crucial pieces of the menopause puzzle, the rich yet distinct histories and backgrounds across women, and safety-related treatment considerations which preclude random assignment, make systematic experimental control for scientific inquiry in women a challenge. For instance, currently available estrogen-containing treatment in the clinic must include an opposing progestogen for a woman that has her uterus to offset the estrogen-associated risk for endometrial hyperplasia and cancer [1]. Thus, experimentally evaluating estrogen-only effects on cognition in menopausal women with an intact uterus is not a safe and viable option, and evaluating estrogen plus progestogen hormone combination effects on cognition does not yield straightoforward information on the individual contribution of each hormone to the exhibited cognitive outcomes.

Because both estrogens and progestogens are such important components to a safe HT picture from a multiple systems perspective, experimentally studying both estrogens and progestogens, alone and in combination, is critical. Generally speaking, based on findings using our behavioral battery: (1) estrogens can enhance learning and memory, especially with particular treatment regimens (17b-estradiol, and delta 8,9 dehydroestrone), while some estrogens can impair learning and memory (estrone, and the synthetic ethinyl estradiol), and (2) natural progesterone and several synthetic progestogens (medroxyprogesterone acetate, norethindrone acetate) can impair memory, and only one synthetic progestogen evaluated so far has revealed enhancing effects (levonorgestrel). Discovering estrogens and progestogens that yield beneficial, or at least neutral, effects on the brain and its functioning will tremendously grow and enrich the breadth of options available for the array of scenarios whereby exogenous hormones are prescribed, spanning not only HTs but also contraceptives as well as treatments for hormone-related health conditions.

Preclinical models of menopause, particularly rodent models, have been instrumental in understanding the effects of ovarian hormone loss on learning and memory, as well as the effects of exogenous hormone administration, across a multitude of parameters [5–7]. Rodent models allow systematic manipulation and evaluation of how incremental changes to one or more of these parameters impact cognition. The overall consensus so far: one plus one does not always equal two; hormones interact in ways that we cannot yet reliably predict. On numerous occasions, we and others have shown that the potent 17b-estradiol can, but does not always, enhance cognition.

We have also discovered that natural progesterone reverses the cognitive benefits and some brain changes induced by 17b-estradiol treatment. At the same time, we were repeatedly finding markedly impairing effects of synthetic progestogens, which was pushing us to delve deeper into other progestogen classes that could yield positive effects on cognition, resulting in us testing a series of progestogens at varied temporal intervals and doses [7]. Thus, after years of finding cognitively-impairing progestogen-induced effects in our laboratory, when we discovered a progestogen, levonorgestrel, that yielded positive cognitive effects when exogenously administered alone, we immediately tested whether 17b-estradiol benefits could withstand concurrent exposure to levonorgestrel [6,7]. Indeed, this was possible, as both 17b-estradiol and levonorgestrel could yield beneficial effects when given alone, and we knew the doses and regimens to use since we found positive effects in our own model and behavioral battery.

Importantly, this assessment was clinically relevant as this combination is used in HT. In our recent study, we replicated the beneficial cognitive effects of 17b-estradiol given alone, and we also replicated the beneficial cognitive effects of levonorgestrel given alone. However, this cognitively beneficial dose of levonorgestrel prevented the cognitively enhancing effects of 17b-estradiol alone, and, relative to each hormone alone, these two hormones were impairing at high working memory demand only when given together [6]. Thus, this eureka moment of finally finding a progestogen that enhanced cognition turned into the realization that while testing hormones individually can yield important discoveries, the real test to truly make a translational impact for HTs will need to include clinically-relevant hormone combinations. Indeed, one plus one does not predictably equal two for hormone combinations. Alternate strategies must be explored, including designing novel HTs focused on targeted delivery, to achieve greater appropriately-regimented estrogen activity in the brain than in the periphery, and greater appropriately-regimented progestogen activity in the uterus than in the brain. To be most efficacious, scientific strategies should be armed with knowledge regarding where and when during a lifetime hormone exposure is optimal, as well as what type and concentration of hormone is most beneficial given specific hormonal historical contexts. Identifying the particular impactful components of these complex interactions is necessary to fill in the missing pieces of the puzzle. This should optimally include basic science and clinical perspectives while using a multi-systems tactic. In taking this approach, many findings that may currently appear contradictory will likely converge. In fact, as we are scientific learners and making discoveries about the truth in nature, it is becoming increasingly apparent that outcomes across studies that appear contradictory, are not contradictory at all. Rather, we are learning of mediating variables that impact the extent and direction of menopause and hormone effects on the brain and cognition. These mediators, most of which are not yet determined or well-understood, will be the puzzle pieces to help fill our gaps in knowledge and give us a more complete picture of optimizing women’s health across a lifetime. Gaining understanding of the rich complexity of women’s experiences to optimize overall health requires intense and systematic inquiry, noting that complexity does not mean impossibility. Undoubtedly, exciting breakthroughs in women’s healthcare during aging are on the horizon, but only with a continued momentum of research. We are fervent on this path, we must push forward with an open mind, and we can do it if we work together crossing disciplines and domains of science spanning applied clinical care to basic benchside discovery.

## References

[1] NAMS. 5th ed. Mayfield Heights: The North American Menopause Society (NAMS); 2014.

[2] Koebele SV, Bimonte-Nelson HA. Exp Gerontol. 2017; 94:14–23. 10.1016/j.exger.2016.12.011 27979770 PMC5479709

[3] Micevych P, Sinchak K. Mol Cell Endocrinol. 2008; 290:44–50. 10.1016/j.mce.2008.04.016 18572304 PMC2603025

[4] NAMS. Menopause J North Am Menopause Soc. 2017; 24:1–26. 10.1097/GME.0000000000000798

[5] Koebele SV, Bimonte-Nelson HA. Maturitas. 2016; 87:5–17. 10.1016/j.maturitas.2016.01.015 27013283 PMC4829404

[6] Prakapenka AV, et al. Neurobiol Aging. 2018; 64:1–14. 10.1016/j.neurobiolaging.2017.11.015 29316527 PMC5820186

[7] Braden BB, et al. Behav Brain Res. 2017; 322:258–68. 10.1016/j.bbr.2016.06.053 27368418 PMC5195920

